# The clinical-radiological paradox in multiple sclerosis: myth or truth?

**DOI:** 10.1055/s-0042-1758457

**Published:** 2023-03-14

**Authors:** Ana Hartmann, Fabio Noro, Paulo Roberto Valle Bahia, Fabricia Lima Fontes-Dantas, Rodrigo Ferrone Andreiuolo, Fernanda Cristina Rueda Lopes, Valeria Coelho Santa Rita Pereira, Renan Amaral Coutinho, Amanda Dutra de Araujo, Edson Marchiori, Soniza Vieira Alves-Leon

**Affiliations:** 1 Universidade Federal do Rio de Janeiro, Departamento de Radiologia, Rio de Janeiro RJ, Brazil.; 2 Universidade Federal do Rio de Janeiro, Departamento de Neurologia, Rio de Janeiro RJ, Brazil.; 3 Universidade Estadual do Rio de Janeiro, Departamento de Farmacologia e Psicobiologia, Rio de Janeiro RJ, Brazil.; 4 Rede Dor, São Luiz, Rio de Janeiro RJ, Brazil.; 5 Universidade Federal Fluminense, Departamento de Radiologia, Rio de Janeiro RJ, Brazil.; 6 Universidade Federal do Estado do Rio de Janeiro, Laboratório de Neurociências Translacional. Soniza Vieira Alves-Leon, Rio de Janeiro RJ, Brazil.

**Keywords:** Multiple Sclerosis, Magnetic Resonance Imaging, Demyelinating Diseases, Esclerose Múltipla, Imageamento por Ressonância Magnética, Doenças Desmielinizantes

## Abstract

**Background** Multiple sclerosis (MS) is an inflammatory, degenerative, demyelinating disease that ranges from benign to rapidly progressive forms. A striking characteristic of the disease is the clinical-radiological paradox.

**Objectives** The present study was conducted to determine whether, in our cohort, the clinical-radiological paradox exists and whether lesion location is related to clinical disability in patients with MS.

**Methods** Retrospective data from 95 patients with MS (60 women and 35 men) treated at a single center were examined. One head-and-spine magnetic resonance imaging (MRI) examination from each patient was selected randomly, and two independent observers calculated lesion loads (LLs) on T2/fluid attenuation inversion recovery sequences manually, considering the whole brain and four separate regions (periventricular, juxtacortical, posterior fossa, and spinal cord). The LLs were compared with the degree of disability, measured by the Kurtzke Expanded Disability Status Scale (EDSS), at the time of MRI examination in the whole cohort and in patients with relapsing-remitting (RR), primarily progressive, and secondarily progressive MS.

**Results** High LLs correlated with high EDSS scores in the whole cohort (r = 0.34; *p* < 0.01) and in the RRMS group (r = 0.27; *p* = 0.02). The EDSS score correlated with high regional LLs in the posterior fossa (r = 0.31; *p* = 0.002) and spinal cord (r = 0.35; *p* = 0.001).

**Conclusions** Our results indicate that the clinical-radiological paradox is a myth and support the logical connection between lesion location and neurological repercussion.

## Resumo

**Antecedentes** A esclerose múltipla (EM) é uma doença inflamatória, degenerativa e desmielinizante que varia de formas benignas a rapidamente progressivas. Uma característica marcante da doença é o paradoxo clínico-radiológico.

**Objetivos** O presente estudo foi realizado para determinar, se na nossa amostragem, o paradoxo clínico-radiológico existe e se a localização das lesões está relacionada à incapacidade clínica em pacientes com EM.

**Métodos** Foram examinados retrospectivamente dados de 95 pacientes com EM (60 mulheres e 35 homens) atendidos em um único centro. Um exame de ressonância magnética de cada paciente foi selecionado aleatoriamente, e dois observadores independentes calcularam as cargas lesionais (CLs) em sequências T2 e FLAIR manualmente, considerando todo o cérebro e quatro regiões separadamente (periventricular, justacortical, fossa posterior e medula espinhal). As CLs foram comparadas com o grau de incapacidade, medido pela Escala de Status expandido de incapacidade (EDSS, na sigla em inglês) de Kurtzke, no momento do exame de ressonância magnética (RM) em toda a coorte e em pacientes com as formas surto remissão (SR), primariamente progressiva (PP), e secundariamente progressiva (SP) da EM.

**Resultados** Cargas lesionais elevadas foram correlacionadas com altos índices de EDSS considerando toda a coorte (r = 0.34; *p* < 0.01) e no grupo SR (r = 0.27; *p* = 0.02). O EDSS foi correlacionado com CLs altas na fossa posterior (r = 0.31 *;* *p* = 0.002) e na medula (r = 0.35; *p* = 0.001).

**Conclusões** Nossos resultados indicam que o paradoxo clínico-radiológico é um mito e apoiam a conexão lógica entre a localização da lesão e a repercussão neurológica.

## INTRODUCTION

Multiple sclerosis (MS) is an inflammatory and neurodegenerative demyelinating disease that affects the central nervous system (CNS). It may vary from heterogeneous phenotypes, which consist of few physical disabilities even after years of having the disease, to the rapidly progressive forms that present significant physical disability since the diagnosis. 1 One of the striking characteristics of the disease is the so-called clinical-pathological paradox. This term was created to describe the discrepancy found in many patients who did not present disease severity but showed a vast number of post-mortem brain lesions. In other patients, the exact opposite occurred. 2

Since the image can represent an in vivo biomarker of the severity of the pathology, a natural derivation arose: the so-called clinical-radiological paradox. 3 4 The MRI findings play an important role in the diagnostic and follow-up of MS patients, having also been used as predictive data of the effect of the treatment on clinical relapses. 5

The objective of the present work is based on the following questions: Does such a clinical-radiological paradox exist? Or is it just a myth? Is the location of the lesions directly related to the clinical manifestations of the disease?

The strategy to answer these questions consisted of selecting a group of patients with MS, choosing a random period in the timeline, and correlating the load lesion (LL) in the T2/FLAIR (fluid-attenuated inversion recovery) sequences, which represents the radiological manifestation of the disease, with the degree of disability of each patient, measured by the Kurtzke Expanded Disability Status Scale (EDSS). 6

The LL was subdivided into total load lesion (TLL) and regional lesion load (RLL), considering periventricular, juxtacortical, posterior fossa, and spinal cord sites, according to the McDonald criteria of 2017. 7

## METHODS

### Patients

We retrospectively analyzed the data of 95 MS patients (60 women and 35 men) who were diagnosed in both clinical and laboratory bases, during their follow-ups at the outpatient clinic and during the periods of hospitalization, occurring in the last 15 years, at the Hospital Universitário Clementino Fraga Filho of the Universidade Federal do Rio de Janeiro (HUCFF-UFRJ, in the Portuguese acronym), Rio de Janeiro, state of Rio de Janeiro, Brazil. All individuals met the 2017 McDonald criteria for MS diagnosis. According to the disease clinical phenotype, MS was classified as relapsing-remitting (RRMS), primarily progressive (PPMS), and secondarily progressive (SPMS).

Magnetic resonance imaging from patients > 71 years old was not included because of the common hyperintense lesions that are the natural processes of aging that could be interpreted as MS lesions.

The National Research Ethics Council approved the present study (No. 1265), and written informed consent was obtained from all participants.

For each patient, a single MRI examination of the skull and spine (neuroaxis) was chosen for comparison with the clinical situation at that moment. The duration of the disease, the interval between the onset of MS symptoms, and the MRI examination was also studied.

All clinical evaluations were performed by the team of neurologists of the HUCFF-UFRJ, using the Kurtzke EDSS as a reference. 6 The information used was extracted from the records of the patients.

### Assessment by magnetic resonance imaging

Magnetic resonance imaging was performed in a 1.5-T (Magneton Avanto; Siemens, Munich, Germany) with a 12-channel head coil using a conventional protocol ( Table 1 ).

**Table 1:** MRI parameters

Sequences		Matrix	FOV	Slice	TR	TE	Flip
Brain	T1 MPR Sag	256 × 256	250	1	1940	295	15
	PD + T2 TSE Ax	320 × 126	230	4	3100	7.3	150
	T2 Flair Sag	256 × 244	230	4	9000	83	180
	T1 SE Ax MT	256 × 144	230	5	505	9	90
	Flair 3D Sag	256 × 218	260	1	5000	418	Empty
	Diffusion	160 × 160	240	5	3500	83	Empty
	T2 TSE Ax	320 × 216	220	3	3700	102	150
	Epi 2D – DTI	160 × 160	240	3	4000	82	Empty
	Swi 3D Ax	256 × 177	230	2	49	40	15
Spine	T1 TSE Sag Cerv	320 × 224	220	3	463	9	132
	T1 TSE Sag Dors	512 × 307	320	35	645	10	150
	Stir Sag Cerv	320 × 256	250	3	4170	87	150
	Stir Sag Dors	320 × 224	320	3.5	5120	86	150
	T2 Med2 Ax Dors	320z224	250	4.5	602	18	30
	T2 Med2 Ax Cerv	320 × 192	200	4	606	18	30
	T2 TSE Sag	320 × 224	220	3	2940	81	150

Abbreviations; Ax, axial; Cer, cervical; Dors, dors; Epi, echo-planar imaging; MPR, multiplanar reformation or reconstruction; PD, proton density; Sag, sagittal; SE, spin echo; Swi, susceptibility weighted imaging; TSE, turbo spin echo.

The presence, size, and location of hyperintense lesions in T2/FLAIR were determined. Following the modified McDonald criteria of 2017, lesion locations were subdivided as periventricular, juxtacortical, posterior fossa, and spinal cord. 7

Two observers with 25 and 10 years of experience, respectively, without access to patient information, counted and measured the lesions by the visual method/manually. The discrepancies were solved by consensus.

**Figure 1.**
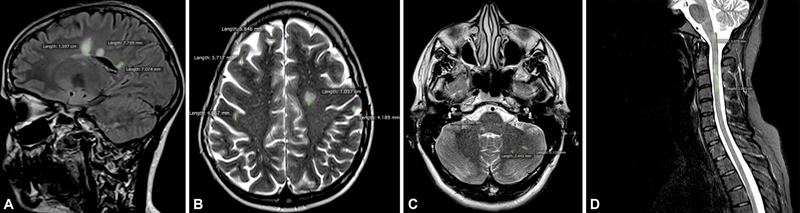
Examples of manual lesion segmentation and measurement in different locations. **A** . Fluid attenuation inversion recovery, periventricular lesions; **B** . Axial T2*-weighted imagens, juxtacortical and periventricular lesions; **C** . Axial T2*-weighted images, posterior fossa; **D** . Sagittal STIR (short T1 inversion recovery), spinal cord. For each lesion, the largest axis was measured (lines).

After this evaluation, hyperintense T2/FLAIR lesions were classified according to size (0–4.9 mm, 5–9.9 mm, 10–19.9 mm, and > 20 mm). Based on the measurement, their estimated mean volumes were determined by considering them as ovaloid figures (0–4.9 mm = 0.01 ml; 5–9.9 mm = 0.27 ml; 10–19.9 mm = 1.76 ml; and > 20mm = 4.18ml). Examples of manual lesion segmentation and measurement in different locations are shown in Figure 1 .

The TLL was calculated by multiplying the number of lesions by their respective estimated average volumes and summing the results. The RLL was calculated separately, according to the locations of the lesions: periventricular, juxtacortical, posterior fossa, and spinal cord. All comparisons of TLL and RLL between the groups were adjusted by age, sex, and duration of the disease.

### Statistical analysis

The database containing patient information was entered in Microsoft Excel (Microsoft Corporation, Redmond, WA, USA) and was subsequently exported to the SPSS Statistics for Windows, version 14.0 (SPSS Inc., Chicago, IL, USA). The data were separated by category, according to the clinical classification of the patients. These data distributed according to proportions was compared using the chi-squared test (Fisher or Yates, depending on the need). Since the present study involves unequal variables, the Tamhane T2 test was performed to compare the mean of the multiple groups. P-values < 0.05 were considered significant.

## RESULTS

### Demographic data

Seventy-three patients presented the clinical forms of RRMS (77%), 9 PPMS (9%), and 13 SPMS (14%). Of the total number of patients, 83.5% (61 patients) were female. Considering the different clinical forms, we found 49 women and 24 men in RRMS, 2 women and 7 men in PPMS, 10 women and 3 men in SPMS. Considering the entire sample, the average time of disease was 17.5 months. Regarding clinical forms, the average time was 17 months in RRMS, 15 months in PPMS, and 21 months in SPMS. The mean age of the patients was 45 years old in RRMS form, 54 years old in PPMS, and 53 years old in SPMS ( Table 2 ).

**Table 2:** Demographics, MRI findings, and clinical data considering the three forms of the disease

Variable		Clinical types			*p* - *value*
		PP	RR	SP	
Patients (%)	–	9 (9.5)	73 (76.8)	13 (13.7)	–
Gender	M (%)	7 (20.0)	24 (68.6)	4 (11.4)	< 0.03
	F (%)	2 (3.3)	49 (81.7)	9 (15.0)	N
Periventricular (LL)	Mean (SD)	16.9(21.9)	12.9(1.5)	32.9(5.5)	N
Periventricular (NL)	Mean (SD)	31(12.2)	38.(26.5)	46.2(22.4)	N
Juxtacortical (LL)	Mean (SD)	10(20.7)	3(3.8)	16.3(45.8)	N
Juxtacortical (NL)	Mean (SD)	27.6(15.2)	26.2(20.3)	31.9(14.3)	N
Posterior Fossa (LL)	Mean (SD)	0.4(0.7)	0.8(1.6)	2.8(3.1)	< 0.05(PP vs SP)
Posterior Fossa (NL)	Mean (SD)	2.6(2.6)	4.3(7.2)	13.5(10.5)	< 0.008 (PP vs SP) and p < 0.03 (SP vs RR)
Optic Nerve	Mean (SD)	5.4 ± 8.0	10.6 ± 47.5	9.8 ± 34.6	N
Spinal Cord (LL)	Mean (SD)	7.1(11.1)	5.1(9.2)	14.7(13.8)	N
Spinal Cord (NL)	Mean (SD)	2.3(2.8)	6.1(17.0)	6.5(5.8)	N
Load Lesion - Total	Mean (SD)	34.1(44.1)	21.9(21.8)	66.8(109)	N
Black Holes	Mean (SD)	4.8(5.9)	2.7(5.3)	16.3(14.5)	< 0.02 (SP vs RR)
Enhanced Lesions	Mean (SD)	4.7(8.5)	0.2(0.7)	0.8(1.5)	< 0.04 (PP vs RR)
EDSS	Mean (SD)	5.1(1.6)	3.3(2.1)	6.8(2.3)	< 0.04 (PP vs RR) and p < 0.0001 (SP vs RR)
Disease duration (years)	Mean (SD)	15.7(14.8)	17.1(11.4)	21.2(13.7)	N
Age at MRI (years old)	Mean(SD)	54.6(12.8)	45.1(13.4)	53.1(16.4)	N

Abbreviations: EDSS, Expanded Disability Status Scale; LL, load lesion; N, Not significant; NL, number of lesions; PP, primary progressive; RR, relapsing-remitting; SP, secondary progressive.

### EDSS-based clinical data

The mean of the EDSS, considering the whole sample, was 3.9. The EDSS mean in clinical forms was: 3.3 in RRMS, 5.1 in PPMS, and 6.8 in SPMS, and a statistically significant difference was observed between RRMS and PPMS clinical forms ( *p* < 0.04) and between RRMS and SPMS ( *p* < 0.001) ( Table 2 ).

### Load lesion measured by MRI

The mean RLL of the posterior fossa, comparing the PPMS and SPMS groups, showed a significant difference ( *p* < 0.05). The mean TLL was 21.9 ml (±21.8) in RRMS, 34.1 ml (±44.1) in PPMS, and 66.8 ml (±109) in SPMS ( Table 2 ). No statistically significant difference in TLL was demonstrated between the three clinical subgroups ( Table 2 ).

### Correlation between EDSS and TLL

The TLL had a statistically significant correlation with EDSS in all patients studied, regardless of the clinical form of the disease (r = 0.34; *p* < 0.01) ( Table 3 ). Analyzing the correlation between TLL and EDSS in the different clinical forms separately, a significant correlation was found only with the clinical form RRMS (r = 0.27; *p* = 0.02) ( Table 3 ).

**Table 3:** Correlation between EDSS and total load lesion, in the whole cohort, and in the three clinical forms of MS

		EDSS		
		RR	PP	SP
All patients	( *n* = 95)	( *n* = 73)	( *n* = 9)	( *n* = 13)
TLL	r = 0.34 (0.01*)	r = 0.27 (0.02**)	r = 0.39 (0.293)	r = 0.21 (0.473)

Abbreviations: EDSS, expanded disability status scale; PP, primary progressive; RR, relapsing-remitting; SP, secondary progressive; TLL, total lesion load.

Notes: Statistics: Spearman's rank correlation coefficient is shown, with the P-value in brackets. *Correlation is significant at the 0.01 level ( *p* -value); **Correlation is significant at the 0.05 level ( *p* -value).

### Correlation between EDSS and RLL

Considering all sampling, regardless of the clinical form, a statistically significant relationship between EDSS and RLL with the posterior fossa was found (r = 0.31; *p* = 0.002), as well as between EDSS and RLL in the spinal cord (r = 0.35; *p* = 0.001) ( Table 4 ). When the RLL (from the posterior fossa and the spinal cord) was compared with the EDSS, in the different clinical forms, only a statistically significant correlation with the clinical form RRMS was observed (in the posterior fossa [ *p* = 0.01] and in the medulla [ *p* = 0.12]) ( Table 4 ).

**Table 4:** Correlation between EDSS and regional lesion load, the whole cohort, and in the three clinical forms of the MS

		EDSS			
		RR		PP	SP
All patients	( *n* = 95)	( *n* = 73)		( *n* = 9)	( *n* = 13)
RLL Spinal Cord	0.351 (0.001)	0.291	(0.012)*	0.325 (0.394)	0.229 (0.453)
RLL Posterior Fossa	0.316 (0.002)	0.370	(0.001)**	0.167 (0.667)	0.272 (0.368)

Abbreviations: EDSS, expanded disability status scale; PP, primary progressive; RLL, regional lesion load; RR, relapsing-remitting; SP, secondary progressive.

Notes: Statistics: Spearman rank correlation coefficient is shown, with the *p* -value in brackets; *Correlation is significant at the 0.05 level ( *p* -value); **Correlation is significant at the 0.01 level ( *p* -value).

## DISCUSSION

### Correlation between EDSS and TLL

In the present study, we found a statistically significant but weak correlation (considering Spearman correlation degrees, ranging from r = 0.3–0.5 [weak] to 0.9–1.0 [very strong]) between the TLL and the EDSS, considering the whole sample (r = 0.34; *p* < 0.01). Several studies in the studied literature show similar results.

A review carried out by Barkoff cites studies in which the degree of correlation found varied from r = 0.15 to 0.69. 4 The author pointed out possible factors that would justify the discrepancy of results in the analysis of the TLL and EDSS ratio, such as the reduction of EDSS variability through the establishment of inclusion criteria for patients in the cohort (in our study, we did not use EDSS degrees in the inclusion criteria).

Studies with a more homogeneous duration of the disease showed a stronger correlation. An example is a study by Morrissey et al., 8 in which the correlation was r = 0.55. In another study, in which the topographic analysis of lesions was performed and LL was measured related explicitly to the corticospinal tract, the correlation obtained was from r = 0.60 to 0.69. 9

Because this is not a prospective study and because we selected a random moment in the timeline of the disease in each patient, the variable duration of illness was not homogeneous, although there was no statistically significant difference of this variable between the different clinical forms studied.

A stronger correlation between TLL x EDSS (r = 0.48) was also found in a study by Schreiber et al. 10 when compared to the present study. However, the mean duration of the disease and EDSS were much higher than ours (13.6 years and 6 years, respectively). Ciccarelli et al., 11 comparing the use of the 3D FLAIR sequence with the T2 sequence, also found a correlation between the TLL and the EDSS (r = 0.53). Their sample, which consisted of 86 patients, presented a homogeneous duration of the disease (this was a prospective study in which the patients were analyzed for 14 years, since the beginning of the disease).

In another prospective study carried out by Brex et al., 12 with a cohort of 71 patients, a moderate correlation (r = 0.60) was also found among the TLL in the first 5 years, with a long-term clinical deficit.

### Correlation between EDSS and LL in different clinical forms

When the TLL was analyzed separately in the different clinical subgroups, a significant correlation was found between the TLL and the EDSS only in the RRMS form. No significant correlation was found in the progressive forms of the disease. A similar result was also found in some studies (Ciccarelli et al. 11 using an *n* = 7 and Molyneux et al., 13 using an *n* = 27). A possible explanation for this would be the low number of patients with PPMS and SPMS forms, both in their respective cohorts and in the sample of the present study.

In a study presented by Ammitzbøll et al., 14 in which a significant correlation was observed between the TLL and EDSS in progressive forms of the disease, the author gathered patients with PPMS and SPMS forms in a single group ( *n* = 93) to obtain a larger cohort of patients, thus generating greater statistical force.

Molyneux et al. 15 demonstrated in a prospective study with > 600 patients presenting the SPMS form that, like in the RRMS form, the correlation between the TLL and the EDSS was also significant. Schreiber, in 2003, found a correlation between TLL and EDSS in the SPMS form ( *n* = 52), whose patients were sufficient for a substantial expression of this correlation.

Nijeholt et al. 16 found a significant correlation between EDSS and MRI findings in their sample. In the SPMS form, the TLL was higher than in the PPMS and RRMS forms, which differs from our results, perhaps due to the difference in the distribution of the number of patients in the clinical subgroups. In this work, the *n* was RRMS = 28, PPMS = 31, and SPMS = 32, while in our work it was RRMS = 73, PPMS = 9, and SPMS = 13.

However, there are also examples in the studied literature that suggest a lack of correlation between the TLL and EDSS. Rocca et al. 17 reported a weak correlation between EDSS and the alterations observed through conventional MRI (T2 lesions and contrast lesions).

Despite the irreversible accumulation of neurological deficit, most patients with the PPMS form showed a relatively low TLL and low disease activity. Rocca reviewed the main results of the quantitative and structural studies of unconventional MRI and concluded that studies with quantitative MRI support the notion that tissue damage (which goes unnoticed in conventional MRI) affects the white matter of normal appearance and the cortex of these patients. 17

### Correlation between EDSS and RLL

We found a significant correlation between RLL and EDSS in the posterior fossa and in the medulla in the analysis of the whole sample, regardless of the clinical subgroup ( Table 4 ).

Several authors, such as Goodin, 18 also found a correlation between the lesions observed in MRI, separated by regions, and the EDSS. The authors used mathematical models to verify this relationship. They considered two types of lesions: "critical," located in the motor fibers of both the internal capsule and the medulla, and "noncritical," located in periventricular regions, corpus callosum, juxtacortical, and deep white matter.

Kerbrat et al. 19 published a study with 290 patients from 8 different centers, where lesions were found along the corticospinal tract, from the cortex to the cervical spinal cord. A significant correlation was demonstrated between the EDSS and the volume of lesions in the corticospinal tract in the brain (r = 0.31; *p* < 0.0001), in the brain stem (r = 0.45; *p* < 0.0001) and in the cervical medulla (r = 0.57; *p* < 0.0001), the latter being the most severe impact on motor deficit in patients with multiple sclerosis.

The analysis within the clinical subgroups shows a significant correlation between EDSS and RLL in the spinal cord and in the posterior fossa only in the RRMS clinical form ( Table 4 ). Possibly, the same explanation for the absence of correlation between the TLL and EDSS serves for the RLL and EDSS; that is, a small number of patients with SPMS and PPMS forms results in the lack of appearance of possible genuine relationships due to the lack of statistical power.

Nijeholt et al., studying 28 patients with the RRMS form, 32 with the SPMS, and 31 with the PPMS, observed that the symptoms associated with spinal cord injuries were more evident in the PPMS and SPMS forms. He concluded that, in the RRMS and SPMS forms, both spinal cord injuries and brain injuries contribute as a tool for monitoring MS. Still, in the PPMS form, the clinicoradiological correlation is weak. 16

Kearney et al. 20 also found a significant correlation between spinal cord injury load and EDSS ( *p* < 0.001) in a study with 120 patients: 34 RRMS, 29 SPMS, 29 PPMS. In contrast to our research, he found a higher lesion load on the spinal cord in progressive forms of the disease when compared with RRMS. Perhaps we did not observe this finding due to the lower number of patients studied. In our study, the lesion load in the posterior fossa was significantly higher in the RRMS form compared with the PPMS form.

Our findings agree with the results of different studies that include MRI lesions located in the posterior fossa and in the spinal cord as a predictive risk for greater disability, especially in cervical topography. The ideal would be to analyze the disease time interval to reach higher levels of disability measured by the EDSS, but even in prospective studies that considered these joint outcome variables, the location of the MRI lesions, such as those here related to greater disability, were equally correlated with predictive disability risk factor.

Despite new quantitative MRI techniques, they have not yet been incorporated into daily clinical practice. T2, FLAIR, and pre- and postcontrast T1-weighted sequences are still the most frequently utilized. 21 Hypersignal lesions on FLAIR and T2-weighted sequences are observed in different clinical forms of the disease, in both early and late stages, and translate into inflammatory edema, demyelination, gliosis, and axonal loss. This range of pathological processes generates the nonspecificity of lesions with a hyper signal on T2-weighted sequences. However, as shown above, even with conventional MRI analysis, we found a significant correlation between MRI and clinical deficit.

A meta-analysis with 18,901 RRMS patients indicates that the effects of a treatment on relapses can be accurately predicted by the effect of that therapy on MRI lesions. 21 Also regarding the importance of MRI in the management of MS, it is important to highlight the association of the findings of imaging with the genetic characteristics of the patients. In their cohort, Noro et al. demonstrated that the presence of the HLA-DQA1*04:01 allele with a higher lesion load on T2/Flair MRI sequences is associated with the risk of greater MS severity. 22

### Study limitations

● This is a retrospective study;
● The lack of clinical and imaging evolution because we chose only a single random moment of the disease of each patient;
● The failure to consider other types of lesions observed in MS, which, in theory, correlate with EDSS, such as atrophy of the brain, and grey matter lesions, which had their importance proven concerning the degree of clinical disability (Rocca, 2016).
● Although the analysis of the imaging parameters was performed by two experienced radiologists, separately, away from clinical information, and in double reading, no automated study was used. The detection and measurement of the lesions were performed manually, and it usually means a difficulty to replicate the study.

In conclusion, the statistically significant correlation between the TLL evaluated by MRI in the CNS and clinical deficit allows us to conclude that the so-called clinical-radiological paradox may be only a myth in our study.

The statistically significant correlation between the brainstem and spinal cord LL and clinical deficit proves the logic between lesion topography and neurological repercussion.

The lack of correlation between the neurological deficit and periventricular and juxtacortical lesions allows us to understand the origin of the paradox myth.
